# Comparison Study of Iron Bioaccessibility from Dietary Supplements and Microencapsulated Preparations

**DOI:** 10.3390/nu11020273

**Published:** 2019-01-26

**Authors:** Malgorzata Anita Bryszewska

**Affiliations:** Institute of General Food Chemistry, Lodz University of Technology, 90-924 Lodz, Poland; malgorzata.bryszewska@p.lodz.pl; Tel.: +48-42-6313425

**Keywords:** iron supplementation, food fortification, encapsulation

## Abstract

Iron deficiency is the most common form of malnutrition. Factors responsible for this so-called “hidden hunger” include poor diet, increased micronutrient needs and health problems such as diseases and infections. Body iron status can be increased by the intake of dietary supplements and fortified food. The aim of the present study was to compare iron bioaccessibility from commercial nutritional supplements and iron microcapsules. A comparison study was performed under conditions mimicking gastric and gastrointestinal digestion. A preparation of encapsulated ferrous sulphate or lactate and vitamin C, in a formula, showed bioaccessibility factors of up to 100% when digested individually, and around 60% in the presence of a food matrix. The degree of oxidation of the ferrous ions differed, depending on the type of preparation, the presence of vitamin C and the food matrix. The highest percentage content of ferrous ion, in the soluble fractions after gastrointestinal digestion, was shown by the preparation containing microencapsulated ferrous lactate or ferrous sulphate and vitamin C. Encapsulation seems to limit the interaction of iron with the food matrix and protect it against oxidation, thus making it more accessible for intestinal uptake.

## 1. Introduction

Iron deficiency is the most prevalent mineral deficiency in the world. It affects between one and a half and two billion people worldwide, in both developed and developing countries, according to World Health Organization estimates [1]. Iron deficiency is a general term for suboptimal levels of iron in the body for health. WHO defined iron deficiency as a condition in which there are no mobilizable iron stores and in which signs of a compromised supply of iron to tissues, including the erythron, are noted [2]. Iron deficiency is associated with diminished work productivity, lower immunity and impaired cognitive development [3]. The more severe stages of iron deficiency are associated with anaemia, that is considered to be present when iron-deficient erythropoiesis occurs; individual haemoglobin levels are below two standard deviations of the distribution mean for haemoglobin in an otherwise normal population of the same gender and age, who are living at the same altitude [2]. Three main reasons for iron deficiency can be identified: inadequate iron intake, compromised bioavailability and increased iron losses. The two main dietary strategies used to combat iron deficiency are supplementation and food fortification. Dietary supplements are foodstuffs containing highly concentrated single or complex substances with nutritional or other physiological effects (such as vitamins or minerals). They are intended for daily consumption in small quantities, in addition to the normal diet. Fortification is the addition of a nutrient or nutrients to foods, which may or may not have been an original component in the food. The fortification strategy is often regarded as the most cost-effective, long-term approach to reducing the prevalence of iron deficiency, with fewer side effects than supplementation [4,5].

For successful iron fortification, the most important factors to consider are: (a) the choice of iron compound (preferably containing ferrous ions); (b) its impact on the taste, appearance and shelf life of the final food product; (c) its interaction with the food matrix; and (d) its absorption [6]. The addition of iron may cause a metallic aftertaste, unacceptable flavour change (the result of the oxidation-mediated rancidity of fats), undesirable colour changes (a result of interaction with anthocyanins, flavonoids and tannins) and degradation of vitamins (notably vitamins C and A) and minerals (in particular, iodine from the oxidation of iodide/iodate to free iodine, which escapes as a gas). Successful fortification combines the delivery of a meaningful level of bioavailable iron with minimal or no sensory changes to the food vehicle.

The bioavailability of iron is dependent on the type of food. Iron absorption may be enhanced by simultaneous intake of vitamin C and animal protein [7,8,9]. Dietary components, such as phytic acid, phenolic compounds and calcium, have inhibitory effects on iron absorption [6,10]. One of the approaches used to increase iron absorption from cereal-based complementary foods is the activation of native cereal phytases or the addition of phytases, which degrade phytic acid [11]. Other strategies to overcome the negative effect of phytic acid include addition of ascorbic acid as an absorption enhancer, or the use of NaFeEDTA (sodium ethylenediaminetetraacetatoferrate(III)) [12,13,14,15]. A relatively new solution, recently implemented in the food industry, is active compound encapsulation [16,17]. This method enables undesirable colour changes to be avoided, and can also prevent the interaction of iron with the food matrix, which may have negative results such as fat oxidation during storage, active compound precipitation and complexation.

The amount of an element that is converted under gastrointestinal conditions into a soluble fraction, potentially available for intestinal absorption, is defined as the bioaccessibility [18]. The accessibility of iron released from food depends on its chemical form and the presence of enhancing or inhibiting factors, which increase or decrease its solubility. Nonheme iron transport across the intestinal membrane involves ions (Fe^2+^ or Fe^3+^). Artificial digestion techniques, which simulate gastrointestinal conditions, are widely used to evaluate the bioaccessibility of bioactive compounds in different types of food product. Typically, in-vitro methods are used to mimic the processes that occur in two (or occasionally three) distinct but linked areas of the human digestive system: the stomach and small intestine (and sometimes the mouth) [19,20].

The aim of the present study was to compare iron bioaccessibility from two preparations: dietary supplements and food fortificants containing microencapsulated ferrous sulphate or ferrous lactate. A comparison study was performed under conditions mimicking gastric and gastrointestinal digestion. The digestions were carried out in the presence of selected food products. The effectiveness of encapsulation was evaluated in terms of its ability to increase Fe bioaccessibility and reduce the interaction of Fe with the food components. The novelty of the present work is to determine the impact of a meat sandwich on iron bioaccessibility and iron speciation analysis in the digestates.

## 2. Materials and Methods

### 2.1. Materials

All reagents used were of reagent grade and, unless otherwise stated, were sourced from Sigma. Selectipur nitric acid was sourced from Lach:ner. Iron standard solution, a Certified Reference Material, was obtained from Merck (Darmstadt, Germany). Milli Q distilled deionised water was used throughout the experiments. All glass and polyethylene labware were soaked in concentrated nitric acid for 24 h and then rinsed with distilled deionised water before use to avoid contamination. Working solutions of enzymes were prepared immediately before use. Dietary supplements were purchased from the local pharmacy; the composition of the tablets is presented in the Table 1. Vitamins expressed as percentage of recommended daily intake in one tablet [21]. The weight of one tablet was 0.98 ± 0.04 g and 0.54 ± 0.02 g for dietary supplements 1 and 2 (Spl1 and Spl2), respectively.

### 2.2. Microencapsulated Iron (Microcapsules)

Encapsulated ferrous sulphate (FS) and ferrous lactate (FL) were prepared by AINIA, Centrer Tecnológico (Valencia, Spain) and EPSA (Valencia, Spain). The method is described in [22]. Briefly, a suspension of thermo-resistant modified starch (TMS) was prepared in deionized water, with a concentration of between 85% and 65% g/g. Ferrous sulphate or ferrous lactate was suspended in the TMS solution, to a concentration of 25% to 35% g/g, and spray-dried (Dryer Mini spray-dryer B-290, Büchi, Switzerland).

### 2.3. Food Samples

Food products were purchased from local stores. Subsamples were made from a breakfast sandwich (Brfs) consisting of 50 g of bread (a bread roll), 20 g of roasted ham (Pamso S.A. Pabiancice, Poland), 5 g of butter and 5 g of butterhead lettuce, or from a bread roll only. The bread roll was made from white, wheat flour, prepared by conventional fermentation. All samples were homogenized. This step of sample preparation was included to mimic food fragmentation during mastication. Subsamples of 1 g were weighed precisely, frozen and stored until use.

Phytic acid content in the bread roll was determined by the HPLC (High Performance Liquid Chromatography)according to the method described by Camire and Clydesdale [23].

### 2.4. Scanning Electron Microscopy (SEM)

Scanning electron microscopy (SEM) of the microcapsules was performed using a Jeol-JCM scanning electron microscope (model JCM-6000 Akishima, Tokyo, Japan 2014). Dried samples were placed in the sample holder covered with double-sided carbon electroconductive tape and then covered with a layer of gold (0.5–1 nm thickness). The images were recorded at differences of acceleration potentials, ranging from 5 kV to 10 kV.

### 2.5. Particle Size Distribution

Particle size distribution of the powders (preparations Mi1-4) was measured by laser diffraction technique with Analysette 22 NanoTec plus (FRITSCH GmbH - Milling and Sizing, Idar-Oberstein, Germany). Laser light scattering was caused by the solid material suspended in distilled water. Disintegration of the solid powders was supported by an ultrasound bath with incorporated stirrer.

### 2.6. Fourier Transform-Infrared Spectroscopy (FT-IR)

The analyses were performed on FS, FL, TMS and preparation of encapsulated iron powders. The IR spectra were obtained on Nicolet 6700 (Thermo-Scientific, Waltham, MA, USA) FT-IR spectrometer, in the 4000–600 cm^−1^ region at 2 cm^−1^ spectral resolution and 64 scans per sample. The background spectra were collected before each sample to eliminate signals of the spectrometer and its environment from the sample spectrum. Spectral data were processed using the software of the spectrophotometer (OMNIC ver. 8.0—Thermo Fisher Scientific Inc., Waltham, MA, USA).

### 2.7. Gastrointestinal Digestion

The procedure used for mimicking digestion in the upper gastrointestinal tract of human adults was a modified version of similar methods described in previous studies [24,25,26]. Digestion was carried out with 1 g subsamples of bread or Brfs. The defrosted samples were placed in a wide-mouthed polyethylene (PE) bottle, to which dietary supplement or microcapsules were added. The dietary supplements were first crushed and added to the Brfs in a ratio of 1:80 (1 tablet to 80 g of the breakfast sandwich). The mass of microcapsules added in each digestion simulation was normalized to the total amount of iron present in the relative supplement (i.e., containing the same iron compound). A volume of 5 mL of HCl with pH 2.0 ± 0.1 was added to each bottle and simulation of gastric digestion was initiated by the addition of 0.5 mL of pepsin solution (2 mg pepsin in 0.1 M sodium hydrogen carbonate and 0.01 M HCl). The bottles were placed in a water bath, with the temperature set to 37 °C with shaking. After 2 h, samples corresponding to the gastric phase (G) were removed and centrifuged for 20 min at 4427 RCF. The liquid phase was decanted into a 15 mL polyethylene tube. The supernatants and the pellets were used for analysis of the iron content.

Hydrolysis was continued with the second set of samples, under conditions simulating those of the small intestine. The acidity of the extracts was adjusted to pH 6.8–7.0 by titration of a saturated solution of sodium hydrogen carbonate. A solution (0.5 mL) of digestive enzyme and bile salts (0.5 mg of pancretin and 3 mg of bile salts per 1 mL) was added. The samples were then incubated at 37 °C in the water bath for a further 2 h. When the second step of hydrolysis was complete, the samples were centrifuged in analogical conditions to those described above, and the soluble fractions were collected into 15 mL polyethylene tubes (GI). The supernatants and the pellets were used for analysis of the iron content. All the experiments had a set of controls that followed the same procedure, consisting of the bread, Brfs, a ferrous salt (ferrous sulphate or ferrous lactate), and an enzyme solution. Bioaccessibility was calculated as follows:

$$\mathrm{Bioaccessible}\;\mathrm{fraction}\;\left(\mathrm{BF}\right)=\frac{\mathrm{Ionic}\;\mathrm{iron}\;\mathrm{content}\;\mathrm{in}\;\mathrm{the}\;\mathrm{gastrointestinal}\;\mathrm{digestate}\;\left[µg\right]}{\mathrm{Total}\;\mathrm{iron}\;\mathrm{content}\;\mathrm{in}\;\mathrm{the}\;\mathrm{introduced}\;\mathrm{sample}\;\left[µg\right]}\times 100\%$$

### 2.8. Measurement of Total Iron

The total concentration of iron in the wet-digested samples was determined using atomic absorption spectrophotometry. The Standard Berghof method for microwave digestion of wheat was applied (Berghof Products and Instruments GmbH Labor Technik, Eningen, Germany). Briefly, 10 cm^−3^ of concentrated nitric acid and 2 cm^−3^ of 30% hydrogen peroxide were added to one supplement tablet, or 1 g of a solid sample or 1.5 ÷ 5 mL of a liquid sample. Closed vessels were used. The microwave oven was set to a three-step digestion program, with the temperature rising to 170 °C and a total digestion time of 25 min. The digested samples were diluted with deionized water and the iron content was determined by Atomic Absorption Spectrometry using a GBC 932 spectrophotometer (GBC Scientific Equipment Pty Ltd., Braeside, Australia), with a hollow cathode lamp for iron, at 248.3 nm. Acetylene and air flow were fine-tuned daily. Standard curves were prepared daily by diluting iron standard reference materials (1000 µg/cm^−3^; JT Baker^®^ Chemicals, Phillipsburg, NJ, USA) with deionized water, to concentrations of between 0 and 10 µg/cm^−3^. A certified reference material, wheat flour—trace elements (NIMGBW10011) (LGC, standards, Dziekanow Lesny, Poland), was used to test the accuracy of the methods. Iron recovery was 97 ± 4%. During analysis, precautions were taken to avoid contamination of the iron samples.

### 2.9. Iron Chemical Speciation

The presence of Fe(II) was determined through the reaction of formation coordinating compound with Ferene-S reagent (3-(2-pyridyl)-5,6-difurylsulfonic acid-1,2,4-triazine sodium salt) under acidic conditions [27]. Stable deep blue complex of Fe(II)-Ferene-S gives absorbance at 593 nm. Determination of Fe(II) and Fe(III) was preceded by the reduction of ferric to ferrous ions at room temperature, using ascorbic acid over 10 min of incubation. The concentration of iron(II) was calculated based on a calibration curve. The response was linear and the correlation coefficients (R^2^) were always greater than 0.996. Typical calibration standards ranging from 5–20 µg Fe/L were prepared from the working standard, prepared daily.

### 2.10. Statistical Analysis

Statistical analysis of the data was performed in R computational language [28]. For post-hoc tests, the package Agricolae: Statistical Procedures for Agricultural Research was used [29]. The data were analysed using a one-way ANOVA (*p* < 0.05) followed by multiple comparison using a Tukey’s test, with significance set at *p* < 0.05.

## 3. Results and Discussion

### 3.1. Iron Content

A wide range of supplements are available for the treatments for iron deficiency. They differ in terms of their iron content, the chemical form of the iron used and their mode of administration (oral or injected) [7]. Iron compounds recommended by the World Health Organization for food fortification include ferrous sulphate, ferrous fumarate, ferric pyrophosphate and electrolytic iron powder. Ferrous sulphate and ferrous lactate were selected for the purpose of the present study. Ferrous sulphate is often used as a reference compound in comparative studies of iron bioaccessibility and bioavailability [30]. Ferrous lactate was selected because of its water-solubility, high relative bioavailability (67), relative low cost and thermal stability [31]. Application of thermo-resistant modified starch as a wall material was due to its resistance to stomach conditions and ability to release the core material from capsules only in the small intestine, in reactions driven by hydrolytic enzymes. Modified starch is a biopolymer that has been widely used as encapsulating material to protect compounds such as pigments, vitamins, microorganisms, probiotics lipids and essential oils, flavours and polyphenols from unfavourable gastric conditions [32]. The microcapsules resistance to high temperature and humidity was tested in conditions simulating the baking process [22]. Under the tested conditions, the microcapsules showed a good wall integrity and very limited iron oxidation [22].

The total iron content in the analysed preparations varied from 4.49 to 11.28 mg Fe/100 g in the case of microcapsules, and 6.56 to 10.44 mg Fe/tablet for the dietary supplements (Table 2). In the dietary supplements, the determined iron content was no more than +7% of the value declared by the producer.

One tablet of Spl1 contains 82% of the recommended daily intake of iron for men (6.56 mg of 8 mg/day RDA) and 36% of that for women (10.44 of 18 mg Fe/day RDA) [32]. One tablet of Spl2 provided 130% and 58% of the RDA for men and women, respectively. Encapsulated preparation Mi1 was the most concentrated iron source, therefore in the smallest mass it can provide the iron amount corresponding to the recommended daily intake.

### 3.2. Morphology of Microcapsules

**Figure 1:**
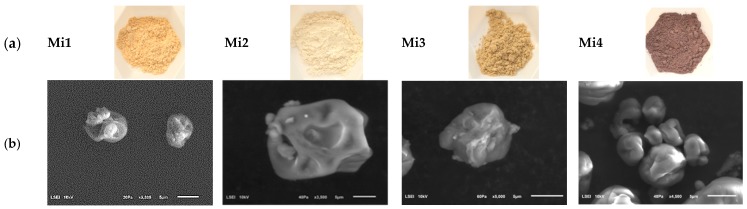
Morphology of microcapsules of iron encapsulated in thermo-resistant modified starch: (**a**) digital images; (**b**) scanning electron microscopic micrographs. FS—ferrous sulphate; FL—ferrous lactate; Mi1—FS; Mi2—FS and Vitamin C; Mi3—FL; Mi4—FL and Vitamin C.

The digital and SEM images of the encapsulated iron preparations are shown in Figure 1. The microcapsules made with FS and vitamin C (25%) have a paler colour than microcapsules containing FS only, indicating that partial oxidation of iron occurred in preparation without vitamin C (Mi2 vs. Mi1). The preparation of microcapsules containing vitamin C at the concentration of 25% and FL (Mi4) had a reddish-brown colour. FT-IR analysis brought explanation of this unexpected colour of Mi4 (see Section 3.3). The surface morphology of the microcapsules was characteristic of those produced by spray drying (Figure 1b). The particles had an amorphous shape, wrinkled with concavities on the surface, as a result of the rapid evaporation of the water. Similar results have been reported by other authors [33].

The microcapsules had a median particle size (d_50_) of less than 80 μm, as determined by laser diffraction. The median particle size was 38.3, 52.3, 62.9 and 75.5 μm for Mi2, Mi4, Mi3 and Mi1, respectively. The particle distributions were unimodal with a weak span, with the exception of duomodal Mi1. Within this range of microcapsule sizes, the preparations could have a broad range of applications. One such application is to fortify wheat flour. Since studied capsules were below 100 µm, they would easily pass through the 150 µm sieves commonly used in the milling industry.

### 3.3. Structural Analysis (FTIR)

**Figure 2:**
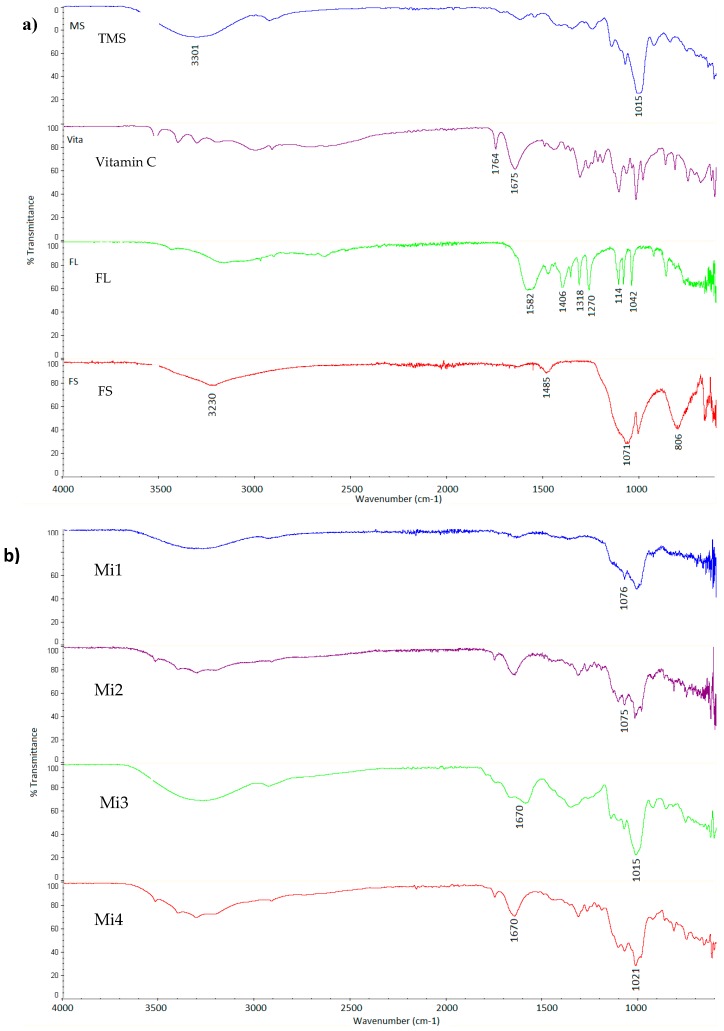
FTIR spectra for: (**a**) components used to build microcapsules (TMS—thermo-resistant modified starch; FS—ferrous sulphate; FL—ferrous lactate); and (**b**) preparations of iron encapsulated in thermo-resistant modified starch (Mi1—FS; Mi2—FS and Vitamin C; Mi3—FL; Mi4—FL and Vitamin C.

FTIR spectra were collected to observe and characterize potential interactions between core (FS, FL, vitamin C) and wall materials (TMS). Figure 2 presents FTIR spectra for the iron microcapsules, as well as for their individual components. The thermo-resistant modified starch showed a strong band at 3301 cm^−1^, which is characteristic for hydroxyl groups [34]. All microcapsules exhibited a similar peak in this functional group region (Figure 2b). However, in the spectra of the preparations containing vitamin C, this band was less evident. The bands for Mi2 and Mi4 remained structurally close to that observed in the spectra of vitamin C. Another important band for the wall material was located at 1015 cm^−1^, which represents C–O bonds stretching vibrations [35]. This seemed to be quite similar in all microcapsules. However, in the microcapsules enhanced with vitamin C, it interfered with the strong band from the ascorbic acid at 1027 cm^−1^.

In the spectra of vitamin C, typical regions of absorbance were observed [36]. The strong bands in the range 3200–3520 cm^−^^1^ in the spectra were indicative of stretching O–H bonds vibrations. The peaks at 1764 and 1675 cm^−^^1^ in the spectrum were attributed to the stretching vibrations in the five-membered lactone ring system. Various bands in the region 1200–1500 cm^−^^1^ were connected with CH_2_ and C–H vibrations and deformation modes. The spectra of the encapsulated preparations revealed small shifts and many overlapping peaks in the regions characteristic for ascorbic acid.

In the spectra, the FL medium and low intense bands observed in the region 1114–1042 cm^−1^ can be ascribed to C–O stretching vibrations [37]. The peaks ranging between 1582–1270 cm^−1^ belong to the asymmetric and symmetric stretching vibrations of COO−functional groups [34]. In the Mi4 microcapsules, the band at 1406 cm^−1^ was reduced. This may be associated with ligand changes in the iron complex. In the salt, iron forms single tooth coordination compounds with lactate anions, but in competition a bond was formed with the ascorbate, as its thermodynamic stability constant is higher [38]. Ligand substitution was also confirmed by the reddish-brown colour of the preparation, which is characteristic for ferrous ascorbate [39].

Four typical bands were observed in the fingerprint region of FS [40]. The main band centred at 1071 cm^−1^, a peak at 3230 cm^−1^, a broad feature at 806 cm^−1^, and a weak peak at 1485 cm^−1^. A band at 806 cm^−1^, characteristic for monohydrate salt, was not observed in the preparations. A main band (1071 cm^−1^) attributed to sulphate groups can be observed in the spectra of both Mi1 and Mi2 with small shifts.

FTIR spectra confirmed that FS, FL and vitamin C were successfully encapsulated within TMS. Spectra of Mi1–Mi3 preserved much of the absorption bands attributed to its components. The spectra revealed differences in Mi4 microcapsules composition. Ferrous ascorbate formedion was suggested, as a result of interaction between vitamin C and ferrous lactate [39,41].

### 3.4. Gastrointestinal Digestion

#### 3.4.1. Total Iron Content in Fractions

Table 3 summarizes the iron content in the supernatants after digestion of the dietary supplements and microcapsules with or without 1 g of food product. Digestions of iron salts (ferrous sulphate and lactate) were performed to evaluate the impact of vitamin C in the preparations and encapsulation on iron solubility.

The amount of iron in the soluble fractions was found to have decreased 2 h after digestion under gastrointestinal conditions. Changes in acidity (from 2.0 to 6.8–7.0) caused increases in the concentration of hydroxide ions, to values high enough to exceed the solubility product constant (K_SP_) of iron(III) hydroxide (6·10^−38^) and probably iron(III) oxide precipitated. The K_SP_ of iron(II) hydroxide could not be exceeded under the conditions of the experiment. This may explain the higher iron content observed in digestates after GI digestion in the preparations containing vitamin C (FL vs. Spl2; Mi1 vs. Mi2; Mi3 vs. Mi4). The presence of the reducing agent protected ferrous ions against oxidation, and the total iron contents of the soluble fractions produced during G and GI were very similar. In the majority of digestates obtained after digestion in the presence of the food matrix, higher iron content was found in the soluble fractions of preparations containing encapsulated iron (gastric digestion: Mi1 and Mi2 vs. Spl1 or FS; Mi3 vs. Spl2 and FL; gastrointestinal digestion: Mi2 vs. Spl1 or FS; Mi4 vs. Spl2 or FL). These results were in agreement with expectations. The thermo-resistant modified starch as the microcapsules’ carrier formed a barrier-limiting possibility for interaction between iron ions and its complexing agents from food. In general, vitamin C in the dietary supplements had no impact on iron solubility (Spl1 vs. FS; Spl2 vs. FL), whereas for microcapsules this impact was positive (the one exception wa Mi and M4 in G fraction). The introduction of a food matrix had a noticeable impact on iron content in both the gastric and gastrointestinal fractions. In the gastric fractions, the iron content in samples with bread was lower or similar to that with Brfs. The results suggest that iron from dietary supplements and microcapsules were immobilized on these food matrices and, in consequence, solubilized to only a limited degree.

Iron availability for absorption from Spl1 or Spl2 estimated based on its content in the soluble fractions would be much lower in comparison to values calculated using total content (Table 1). Iron immobilization can diminish the amount of iron available for intake. In the form of lactate, one tablet of dietary supplements can provide around 56% of the 10 mg/day of iron recommended for men and 31% of the 18 mgFe/day recommended for women (the values in Table 3 should be multiplied by 80 to calculate how much iron can be absorbed in total from one bread roll or Brfs—multiplied by 80 to calculate total iron uptake from one bread roll or Brfs [32]. In the form of sulphate, one tablet of the dietary supplement can provide around 25% of the RDA for men and 14% of that for women. The daily requirement can be satisfied most effectively by microencapsulated FS and vitamin C (Mi2) in the absence of a food matrix.

#### 3.4.2. Iron Bioaccessiblity

In this study, the effects of a food matrix on the accessibility of iron from dietary supplements and microcapsules were investigated using a breakfast sandwich, chosen because it was assumed that the preferred time of the day for taking dietary supplements is in morning, with the first meal. The masses of the individual products used to prepare the sandwiches were consistent with the nutritional values of the selected foods and typical dishes [42]. Table 3 shows the bioaccessibility of iron (BF) from the soluble fractions obtained after gastrointestinal digestion.

The BF of the supernatants after gastric digestion of dietary supplements and microcapsules was over 87%. Lower BFs were observed for samples containing reference salts: 81% for ferrous sulphate and 71% for ferrous lactate (data not shown). Transfer to intestinal conditions was accompanied by a decrease in BF. This tendency was observed for all samples. The drop in BF was particularly evident when iron was introduced in the form of ferrous lactate, decreasing to 12.8% (Mi3). Under GI conditions, presence of vitamin C effectively prevented the loss of BF (12.8% vs. 106.5% for Mi3 and Mi4). The BF of the digestate of Spl1 containing ferrous sulphate and vitamin C was similar to the reference FS and half that observed for Mi2.

The addition of a food matrices to dietary supplements did not change the BF of the GI digestates significantly, regardless of whether it was bread or Brfs. The iron bioaccessibility was significantly lower in the digestates of food matrices with supplements then in corresponding microcapsules (Spl1 vs. Mi2; Spl2 vs. Mi4).

The addition of food to microcapsules had a significant influence on BF. An increase of the bioaccessibility factor in case of Mi1 and Mi3 may seem paradoxical, but it resulted from enzymatic activity of amylase from pancreatin. In the absence of starch from bread, TMS was hydrolysed in much higher extent. Iron released from the microcapsules underwent oxidation and precipitation from the solution. The significantly lower iron bioaccessibility observed in the digestates of Mi2 and Mi4 with a food matrices was probably associated with the immobilization of the iron by phytic acid. It is well known that phytic acid is a food inhibitor, which chelates micronutrients including iron, reducing their bioavailability [6,11]. Phytic acid content in bread rolls was 2.98 mg/g, which corresponds to an average value. Most of phytic acid concentrations are with the range 2–4 mg/g for the refined wheat flours [43]. Only in the case of BF from Mi1; however, was there a statistically significant difference between the results of adding bread or Brfs. The combination of iron bioaccessibility enhancer (meat) and its inhibitors (phytic acids) increased the BF; however, the change was not statistically significant.

Iron bioaccessibility from dietary supplements and microcapsules, digested individually (without food products), was not influenced significantly by the chemical form of the iron in the preparations (Spl1 and Spl2; Mi1 and Mi3; Mi2 and Mi4). Significant differences in BF were observed between microcapsules when vitamin C was included in the formulations: (Mi1 vs. Mi2; Mi3 vs. Mi4). The impact of vitamin C was also observed when the preparations were digested with bread. The BF of the preparation containing FS with vitamin C was 59.4% (Mi2), compared to 45% (Mi1) in the FS preparation without vitamin C. In the case of the preparation containing FL, BF increased with vitamin C by between 49.3% (Mi3) and 59.0% (Mi4). The antioxidant agent protected the ferrous ions against oxidation and in consequence precipitation of iron(III) hydroxide.

Differences in BF were no longer observed when the preparations were digested with Brfs. There were no significant differences (*p* > 0.05) in BF either between microcapsules (Mi1-Mi4) or microcapsules (Mi1-Mi4) and Spl2. Only Spl1, containing FS, differed. The BF was significantly lower in the digestates of Spl1 with both bread and with Brfs. The results are in agreement with results obtained in the experiments with Caco-2 cells [12]. Neither the mRNA expression of DMT1 nor that of IREG1 showed statistical significance between type of iron source.

The bioaccessible fraction of iron in wheat flour and cereals generally is low [3,5]. The high phytate level of cereals is thought to be responsible for this low iron bioavailability. Encapsulation seems an effective way of limiting iron immobilization by phytic acid and of increasing its accessibility for uptake.

#### 3.4.3. Iron Speciation

**Figure 3:**
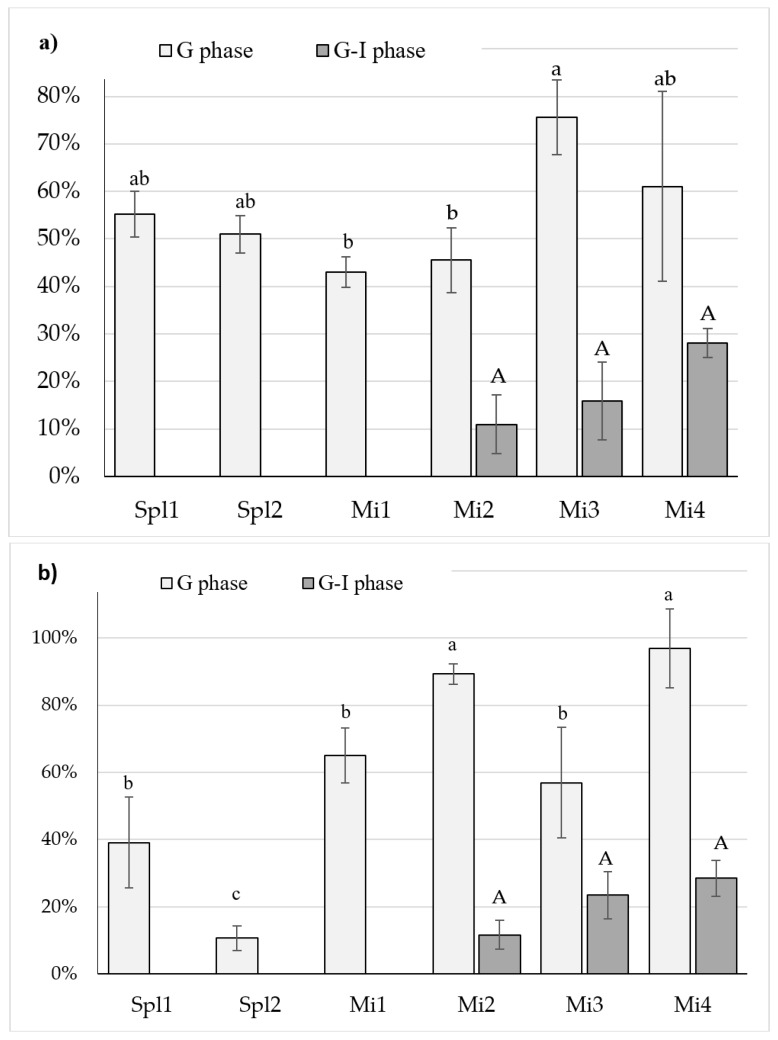
Percentage content of Fe^2+^ in the digests of: (**a**) bread; and (**b**) breakfast sandwich in the presence of preparations (Spl1 and Spl2—dietary supplements; Mi1—Mi4 fortifcants of encapsulated iron) after simulated gastric and gastrointestinal digestion. The content was expressed as the percentage of Fe^2+^ relative to total ionic iron content (Fe^2+^ + Fe^3+^). Results are presented as the means ± SD; *n* = 3. Values with the same letter are not significantly different *p* < 0.05. a, b, c—Letters represent differences between digestates; capital (A): G-I- phase, lowercase (a, b, c): GI- phase

The form of iron affects its passage through the enterocyte and into the circulation around the body. The first nonheme iron acceptor on brush-border membranes is a transporter of divalent ions. This selective mechanism of transport requires the reduction of ferric ions, and makes the absorption of ferrous ions much more efficient. In the present study, in addition to determining the content of soluble iron in the digestate, the ratio of divalent ions to total soluble iron was analysed. Figure 3 presents the percentage content of ferrous ions in the digestates of preparations with bread and Brfs. In the soluble fraction after gastric digestion of dietary supplements or microcapsules with bread, ferrous ions accounted for between 43% and 76% of the iron (Mi1 and Mi3, respectively). Continued digestion under intestinal conditions caused further oxidation, until finally divalent ions were not detected in the digestates of dietary supplements and Mi1. The ferrous ion content in digestates after gastric digestion of dietary supplements and microcapsules in the presence of Brfs varied more widely, between 11% and 97% (Spl2 and Mi4, respectively). In samples containing bread, iron was completely oxidized after gastrointestinal digestion in the digestates of dietary supplements and Mi1. Despite the presence of the reducing agent in the formula of Spl1 and Spl2, complete iron oxidation occurred. There were no statistical differences in the ferrous ion content in GI digestates with a food matrix. Nearly 30% of the iron introduced as ferrous lactate in the form of microcapsules (Mi4) stayed in the divalent form after 4 h of digestion, compared to 28% in the digestates containing bread and 29% in the digestates containing Brfs.

## 4. Conclusions

All the analysed preparations showed high bioaccessibility, measured in terms of iron content in the soluble fraction. However, speciation analysis showed that only in the case of preparations with microcapsules was the iron available in the easily accessible ferrous form. In studies of iron bioaccessibility, speciation analysis should be performed to avoid the inclusion of ferric ions that do not pass through the intestinal membrane, and may thus not be directly bioaccessible. Encapsulation seems to limit the interaction of iron with food matrices and to protect it against oxidation, making it more accessible for intestinal uptake. The type of iron source, ferrous sulphate or ferrous lactate, in the microcapsules had no statically significant impact on its bioaccessibility. 

## Figures and Tables

**Table 1:** Composition of dietary supplements (Spl1 and Spl2).

Vitamins			Mineral Components		
	Spl1 [%]	Spl2 [%]		Spl1	Spl2
A	100.0	np	Iron		
B_1_ Thiamin	90.9	91.0	Ferrous sulphate	6.15 mg	np
B_2_ Riboflavin	100.0	93.0	Ferrous lactate	np	9.9 mg
B_5_ (Pantothenic acid)	16.6	113.0	Zinc	3.00 mg	10.6 mg
Niacin	62.5	74.0	Fluoride	0.525 mg	np
B_6_	15.0	103.0	Manganese	0.33 mg	np
B_12_	100.0	32.0	Copper	0.2 mg	np
C	50.0	50.0	Iodine	50.0 µg	100.0 µg
D	100.0	np	Molibdenium	7.5 µg	np
E	50.0	55.0			
Folic acid	100.0	132.0			
Biotin	np	200.0			

Vitamins expressed as percentage of recommended daily intake in one tablet, based on Regulations of the Health Minister, 2010 DzU 16, poz. 89 [21]. np—not present—a compound not included in the composition of the dietary supplement.

**Table 2:** Total iron and vitamin C content in preparations (Spl1 and Spl2—dietary supplements; Mi1—Mi4 fortificants of encapsulated iron) containing ferrous sulphate (FS) or ferrous lactate (FL). Results are presented as the means ± SD; *n* = 3.

Preparation	Iron Source	Vitamin C	Fe
		(g/g)	mg/100 g
Mi1	FS	n.a.	11.28 ± 0.49
Mi2	FS	0.25	8.28 ± 0.38
Mi3	FL	n.a.	5.87 ± 0.15
Mi4	FL	0.25	4.49 ± 0.08
		mg/tablet	mg/tablet
Spl1	FS	40.0	6.56 ± 0.35
Spl2	FL	39.6	10.44 ± 0.08

**Table 3:** Total iron content in the soluble fractions after gastric (G) and gastro-intestinal (GI) digestion (part A) and the bioaccessible fractions (BF %) (part B), from preparations (Spl1 and Spl2—dietary supplements; Mi1-Mi4 fortificants of encapsulated iron) digested individually or in the presence of a food matrix (white wheat bread or breakfast sandwich (Brfs)).

	Variants of Digestion					
	Preparations		Preparations with Bread		Preparations with Brfs	
	G	GI	G	GI	G	GI
**Part (A)**						
	**µg**	**µg**	**µg**	**µg**	**µg**	**µg**
Ferrous sulphate	104.6 ± 0.4	47.0 ± 11.5	nd	nd	nd	nd
Ferrous lactate	109.7 ± 4.4	22.9 ± 1.6	nd	nd	nd	nd
Spl1	75.1 ± 4.1	29.2 ± 12.3	27.7 ± 3.7	30.8 ± 3.3	11.3 ± 5.9	30.6 ± 5.3
Spl2	109.5 ± 0.2	66.2 ± 0.7	35.9 ± 15.9	70.0 ± 7.5	16.7 ± 3.9	67.2 ± 8.3
Mi1	112.4 ± 1.1	31.9 ± 4.6	24.0 ± 2.5	45.5 ± 3.9	34.4 ± 0.8	71.5 ± 0.7
Mi2	123.9 ± 4.5	123.5 ± 6.2	31.4 ± 6.2	69.4 ± 1.2	45.0 ± 0.5	79.7 ± 4.6
Mi3	178.2 ± 2.3	17.5 ± 3.2	36.2 ± 5.3	89.9 ± 3.3	15.5 ± 0.0	94.9 ± 6.8
Mi4	107.2 ± 9.8	105.3 ± 12.9	44.2 ± 14.2	89.1 ± 1.9	28.3 ± 5.1	70.6 ± 6.7
**Part (B)**						
Spl1		39.2 ^aBC^		31.9 ^aC^		38.0 ^aB^
Spl2		50.6 ^aB^		52.2 ^aAB^		51.6 ^aA^
Mi1		24.6 ^cCD^		45.0 ^bB^		53.6 ^aA^
Mi2		92.9 ^aA^		59.4 ^bA^		57.5 ^bA^
Mi3		12.8 ^bD^		49.3 ^aB^		54.3 ^aA^
Mi4		106.5 ^aA^		59.0 ^bA^		61.1 ^bA^

Results are presented as the means ± SD; *n* = 3. Means with different superscripts differ (*p* < 0.05). Lowercase superscript (a, b, c) represents differences between the rows (comparison of BF from one iron source depends on the digestion conditions). Capital superscripts (A, B, C, D) represent differences within columns (iron sources and presence of vitamin C). nd—digestion was not performed.
